# An empirical study of the BOPPPS teaching model in standardized training for vascular surgery residents: teaching of diabetic foot Wagner classification and surgical intervention strategies

**DOI:** 10.3389/fmed.2026.1804619

**Published:** 2026-04-15

**Authors:** Duan Liu, Jianming Guo, Lianrui Guo

**Affiliations:** Department of Vascular Surgery, Xuanwu Hospital, Capital Medical University, Beijing, China

**Keywords:** BOPPPS teaching model, diabetic foot, resident education, standardized training, vascular surgery, Wagner classification

## Abstract

**Background:**

Diabetic foot ulcers represent a significant clinical challenge requiring comprehensive knowledge of classification systems and surgical intervention strategies. The BOPPPS (Bridge-in, Objective, Pre-assessment, Participatory learning, Post-assessment, Summary) teaching model has gained increasing attention in medical education, yet its effectiveness in vascular surgery residency training remains underexplored.

**Objective:**

This study aimed to evaluate the effectiveness of the BOPPPS teaching model in standardized training for vascular surgery residents regarding diabetic foot Wagner classification and surgical intervention strategies.

**Methods:**

This retrospective cohort study included 196 vascular surgery residents who underwent standardized training at the Department of Vascular Surgery, Xuanwu Hospital, Capital Medical University from January 2023 to December 2024. Based on their training rotation schedule, residents were allocated to either an experimental group (*n* = 98) receiving BOPPPS-based instruction or a control group (*n* = 98) receiving traditional teaching methods. Primary outcomes included theoretical examination scores, clinical skill assessment (Mini-CEX and DOPS), self-directed learning readiness (SDLRS), critical thinking disposition (CTDI-CV), and teaching satisfaction.

**Results:**

The experimental group demonstrated significantly higher theoretical examination scores (82.47 ± 8.63 vs. 74.85 ± 9.21, *p* < 0.001) and Mini-CEX scores (7.84 ± 1.12 vs. 6.93 ± 1.28, *p* < 0.001) compared to the control group. DOPS scores for wound debridement (8.12 ± 0.95 vs. 7.23 ± 1.18, *p* < 0.001), vascular assessment (7.96 ± 1.08 vs. 7.14 ± 1.25, *p* < 0.001), and amputation level determination (7.78 ± 1.15 vs. 6.89 ± 1.32, *p* < 0.001) were significantly improved in the BOPPPS group. Self-directed learning readiness scores increased significantly in the experimental group (168.52 ± 18.74 vs. 153.28 ± 21.36, *p* < 0.001), and critical thinking disposition scores were notably higher (292.84 ± 28.65 vs. 271.35 ± 31.42, *p* < 0.001). Teaching satisfaction rates reached 94.9% in the experimental group versus 78.6% in the control group (*p* < 0.001).

**Conclusion:**

The BOPPPS teaching model significantly enhances the effectiveness of standardized training for vascular surgery residents in diabetic foot management, improving theoretical knowledge, clinical competence, self-directed learning ability, and critical thinking disposition.

## 1 Introduction

Diabetic foot ulcers represent one of the most devastating complications of diabetes mellitus, affecting approximately 15–25% of patients with diabetes during their lifetime and contributing to substantial morbidity, mortality, and healthcare expenditure worldwide (1). The global burden of diabetic foot disease has escalated dramatically, with the International Diabetes Federation estimating that a lower limb is amputated every 20 s due to diabetes-related complications (2). In China, the rapid increase in diabetes prevalence has correspondingly elevated the incidence of diabetic foot complications, creating an urgent need for well-trained vascular surgery specialists capable of providing comprehensive care for this complex patient population (3). The management of diabetic foot ulcers requires a multifaceted approach encompassing accurate classification, appropriate wound care, infection control, revascularization procedures, and timely surgical intervention, making it essential for vascular surgery residents to acquire both theoretical knowledge and practical skills during their standardized training (4).

The Wagner classification system, introduced by F. William Wagner in 1981, remains the most widely utilized grading system for diabetic foot ulcers due to its simplicity and clinical applicability (5). This classification stratifies diabetic foot lesions into six grades (0–5) based on ulcer depth and the presence of infection or gangrene, providing a framework for treatment planning and prognostic assessment. While other systems, such as the University of Texas (UT) classification which incorporates ischemia and infection, or the PEDIS system, offer different perspectives, the Wagner system was selected for this educational intervention because of its foundational role in clinical practice, ease of understanding for novice learners, and its direct correlation with the progression from conservative to surgical management. Despite criticisms regarding its exclusion of vascular status from the formal grading criteria, Wagner’s original treatment algorithms incorporated comprehensive vascular assessment using ankle-brachial index measurements, a principle that was emphasized during the training (6). Understanding the nuances of each Wagner grade and the corresponding surgical intervention strategies is fundamental for vascular surgery residents, as inappropriate treatment decisions may lead to delayed healing, progressive infection, unnecessary amputation, or even mortality (7). However, traditional didactic teaching methods often fail to adequately prepare residents for the complex clinical decision-making required in diabetic foot management, highlighting the need for innovative pedagogical approaches (8).

Standardized residency training programs have been established in China to ensure consistent quality in physician education and competency development across medical specialties (9). For vascular surgery residents, the standardized training curriculum must address a broad spectrum of conditions, with diabetic foot disease representing a particularly challenging area due to its multidisciplinary nature and the diverse range of surgical procedures involved (10). Traditional teaching approaches, characterized by lecture-based instruction and passive learning, have demonstrated limitations in fostering clinical reasoning skills, procedural competence, and self-directed learning abilities essential for effective diabetic foot management (11). The increasing complexity of modern vascular surgery, combined with the expectation that residents achieve proficiency in both open surgical and endovascular techniques, necessitates the implementation of evidence-based educational strategies that maximize learning efficiency and clinical preparation (12).

The BOPPPS teaching model, originating from the Instructional Skills Workshop developed at the University of British Columbia, has emerged as a structured instructional framework designed to enhance student engagement, promote active participation, and provide systematic feedback (13). This model divides the teaching process into six interconnected components: Bridge-in (capturing learner attention and connecting to prior knowledge), Objective (establishing clear learning goals), Pre-assessment (evaluating baseline knowledge), Participatory learning (engaging learners through interactive activities), Post-assessment (evaluating learning achievement), and Summary (consolidating key concepts) (14). Its alignment with constructivist learning theory, which posits that learners actively construct knowledge through interaction, makes it particularly suitable for teaching complex clinical topics like diabetic foot management, where applying knowledge to new situations is critical (15). Recent systematic reviews and meta-analyses have demonstrated the effectiveness of BOPPPS-based instruction in improving academic performance, clinical skills, and learning motivation across various medical education contexts, including pathology, nursing, and internal medicine (16).

However, existing studies on BOPPPS in medical education have several limitations. Many are conducted in classroom-based, pre-clinical settings and rely heavily on short-term knowledge assessments (17). Few have rigorously evaluated its impact on higher-order cognitive skills, such as clinical decision-making, or on professional attributes like self-directed learning and critical thinking disposition in a complex, real-world clinical specialty (18). Furthermore, its application in the demanding field of vascular surgery, which requires the integration of multifaceted skills for conditions like diabetic foot, remains a significant gap.

Despite the growing body of evidence supporting the utility of the BOPPPS model in medical education, its application in vascular surgery residency training, particularly for specialized topics such as diabetic foot management, remains largely unexplored. Traditional lecture-based methods often fail to actively engage residents or adequately develop the complex decision-making skills required for diabetic foot care, creating a critical need for more effective pedagogical strategies. The unique characteristics of vascular surgery education, including the integration of clinical assessment, diagnostic imaging interpretation, wound classification, revascularization techniques, and amputation surgery, present both challenges and opportunities for implementing structured pedagogical frameworks. Furthermore, the assessment of educational outcomes in this context requires consideration of multiple domains, including theoretical knowledge, procedural skills, clinical reasoning, self-directed learning readiness, and critical thinking disposition. Therefore, this study aims to fill this gap by prospectively evaluating the effectiveness of a BOPPPS-based teaching intervention in this specialized context, using a multi-faceted assessment approach.

The primary objective of this study was to compare the theoretical knowledge and clinical competence of vascular surgery residents who received BOPPPS-based instruction versus traditional teaching for diabetic foot management. Secondary objectives were to evaluate the impact of the BOPPPS model on residents’ self-directed learning readiness, critical thinking disposition, and overall teaching satisfaction.

## 2 Methodology

### 2.1 Study design and setting

This prospective quasi-experimental study was conducted at the Department of Vascular Surgery, Xuanwu Hospital, Capital Medical University, which serves as a national standardized residency training base in China. A quasi-experimental design was chosen because randomization of residents to different teaching methods within the same training program was logistically unfeasible, as it would have disrupted the established rotation schedules and could have led to contamination between groups. This design allowed for a rigorous comparison of two distinct educational interventions in a real-world training environment while maintaining the integrity of the program’s structure. The study period spanned from January 2023 to December 2024. The research protocol was approved by the Institutional Ethics Committee, and informed consent was obtained from all participants prior to their inclusion in the study.

### 2.2 Participants

Participants were vascular surgery residents enrolled in the standardized training program who rotated through the diabetic foot management module. Inclusion criteria were: (1) residents in their second or third year of standardized training; (2) completion of basic vascular surgery rotations; (3) willingness to participate in the study; and (4) completion of all assessment components. Exclusion criteria included: (1) previous formal training in diabetic foot management; (2) incomplete attendance (>20% absence) during the training period; and (3) withdrawal from the residency program during the study period.

Sample size calculation was performed using G*Power 3.1.9.7 software. Based on previous studies examining BOPPPS effectiveness in medical education, an effect size of 0.5 was anticipated for the primary outcome measure. With *α* = 0.05, power = 0.80, and an allocation ratio of 1:1, a minimum of 64 participants per group was required. Accounting for a potential 15% dropout rate, we aimed to recruit at least 75 participants per group. Group allocation was determined by the residents’ scheduled training rotation. Residents rotating through the module from January to December 2023 were assigned to the control group and received traditional teaching, while those rotating from January to December 2024 were assigned to the experimental group and received the BOPPPS-based instruction. This non-randomized allocation was based on the training schedule and was not influenced by the researchers or participants. Crucially, the residents in both groups were at similar stages of their training (years 2 and 3) and had comparable baseline characteristics, mitigating potential selection bias. The final sample included 196 residents, with 98 in the experimental group (BOPPPS) and 98 in the control group (traditional teaching).

### 2.3 Intervention

#### 2.3.1 Experimental group (BOPPPS teaching model)

The BOPPPS-based curriculum for diabetic foot Wagner classification and surgical intervention strategies was implemented as follows. The entire training module on diabetic foot management was delivered over a two-week period, comprising four teaching sessions. Each session lasted approximately 90 min and followed the structured BOPPPS format outlined below. The schedule was consistent for all resident rotations in the experimental group.

*Bridge-in (5–8 min):* Each session began with clinical case scenarios, provocative questions, or brief video clips depicting diabetic foot complications to capture residents’ attention and establish clinical relevance. For example, images of different Wagner grade lesions were presented with questions about initial management approaches.
*Objective (3–5 min):* Clear, measurable learning objectives were explicitly stated at the beginning of each session. Objectives were framed using Bloom’s taxonomy and included knowledge, comprehension, application, and analysis levels relevant to Wagner classification and surgical decision-making.
*Pre-assessment (5–10 min):* Baseline knowledge was evaluated through interactive polling, brief written quizzes, or group discussions to identify knowledge gaps and tailor subsequent instruction. Pre-assessment results informed the depth and focus of participatory learning activities.
*Participatory Learning (30–45 min):* This core component employed multiple interactive strategies including case-based discussions, small group problem-solving exercises, standardized patient simulations, surgical video analysis, and hands-on wound assessment workshops. Residents actively practiced Wagner classification on clinical photographs and developed treatment algorithms through collaborative learning.
*Post-assessment (10–15 min):* Learning achievement was evaluated through case analysis exercises, multiple-choice questions, or skills demonstrations. Immediate feedback was provided to reinforce correct understanding and address misconceptions.
*Summary (5–8 min):* Key concepts were consolidated through instructor-led review, resident-generated summaries, or concept mapping exercises. Take-home learning points and clinical pearls were emphasized.

#### 2.3.2 Control group (traditional teaching)

The control group received conventional lecture-based instruction covering identical content regarding diabetic foot Wagner classification and surgical intervention strategies. To ensure content equivalence with the experimental group, the same four-session, 90-min structure was used. The sessions were delivered by the same pool of senior vascular surgery faculty who also taught the experimental group, ensuring comparable instructor expertise. Teaching sessions consisted of traditional didactic presentations using PowerPoint slides, followed by a question-and-answer period. Practical skills training for procedures like wound debridement and ABI measurement was conducted through instructor demonstration, after which residents had the opportunity to practice without the structured, interactive components (e.g., small group problem-solving, standardized patient simulations) that defined the BOPPPS approach.

### 2.4 Assessment instruments

*Theoretical examination*: A standardized written examination consisting of 50 multiple-choice questions and 5 short-answer case scenarios was developed by a panel of senior vascular surgeons. The examination covered Wagner classification criteria, wound assessment, infection grading, vascular evaluation, revascularization indications, and amputation principles. The maximum score was 100 points.

*Mini-Clinical Evaluation Exercise (Mini-CEX)*: Clinical competence was assessed using the validated Mini-CEX tool, which evaluates seven domains: medical interviewing skills, physical examination skills, humanistic qualities, clinical judgment, counseling skills, organization/efficiency, and overall clinical competence. Each domain was rated on a 9-point scale (1–3: unsatisfactory, 4–6: satisfactory, 7–9: superior). The Cronbach’s alpha for Mini-CEX in this study was 0.89.

*Direct observation of procedural skills (DOPS)*: Procedural competence was evaluated through DOPS assessments for three key procedures: diabetic foot wound debridement, lower extremity vascular assessment (including ABI measurement), and amputation level determination. Each procedure was rated on a 9-point scale across six dimensions: procedural indications, informed consent, technical ability, aseptic technique, post-procedure management, and overall performance. The Cronbach’s alpha for DOPS was 0.91.

*Self-Directed Learning Readiness Scale (SDLRS)*: Self-directed learning readiness was measured using the Chinese version of the SDLRS-58, which comprises 58 items across three subscales: self-management, desire for learning, and self-control. Items are rated on a 5-point Likert scale (1 = almost never true to 5 = almost always true). Total scores range from 58 to 290, with higher scores indicating greater self-directed learning readiness. The Cronbach’s alpha was 0.93.

*Critical Thinking Disposition Inventory—Chinese Version* (CTDI-CV): Critical thinking disposition was assessed using the CTDI-CV, a 70-item instrument measuring seven subscales: truth-seeking, open-mindedness, analyticity, systematicity, self-confidence, inquisitiveness, and cognitive maturity. Items are rated on a 6-point Likert scale. Total scores range from 70 to 420, with scores ≥280 indicating positive critical thinking disposition. The Cronbach’s alpha was 0.91.

*Teaching Satisfaction Questionnaire*: A 15-item questionnaire was developed to assess satisfaction with teaching methodology, including content relevance, instructor effectiveness, learning engagement, skill development, and overall satisfaction. Items were rated on a 5-point Likert scale, with total scores ≥60 (out of 75) indicating high satisfaction.

### 2.5 Data collection

Demographic data were collected at baseline, including age, gender, educational background, prior clinical experience, and training year. Theoretical examinations and satisfaction surveys were administered at the conclusion of the training module. Mini-CEX and DOPS assessments were conducted by trained faculty evaluators blinded to group allocation. SDLRS and CTDI-CV were administered before and after the training intervention.

### 2.6 Statistical analysis

Statistical analyses were performed using SPSS version 26.0 (IBM Corp., Armonk, NY, United States). Continuous variables were expressed as mean ± standard deviation (SD) and compared between groups using independent samples *t*-tests or Mann–Whitney *U*-tests, as appropriate based on normality testing (Shapiro–Wilk test). Categorical variables were expressed as frequencies and percentages and compared using chi-square tests or Fisher’s exact tests. Within-group changes in SDLRS and CTDI-CV scores were analyzed using paired *t*-tests. Effect sizes were calculated using Cohen’s *d* for continuous outcomes. A two-tailed *p*-value <0.05 was considered statistically significant.

## 3 Results

### 3.1 Participant characteristics

**Figure 1:**
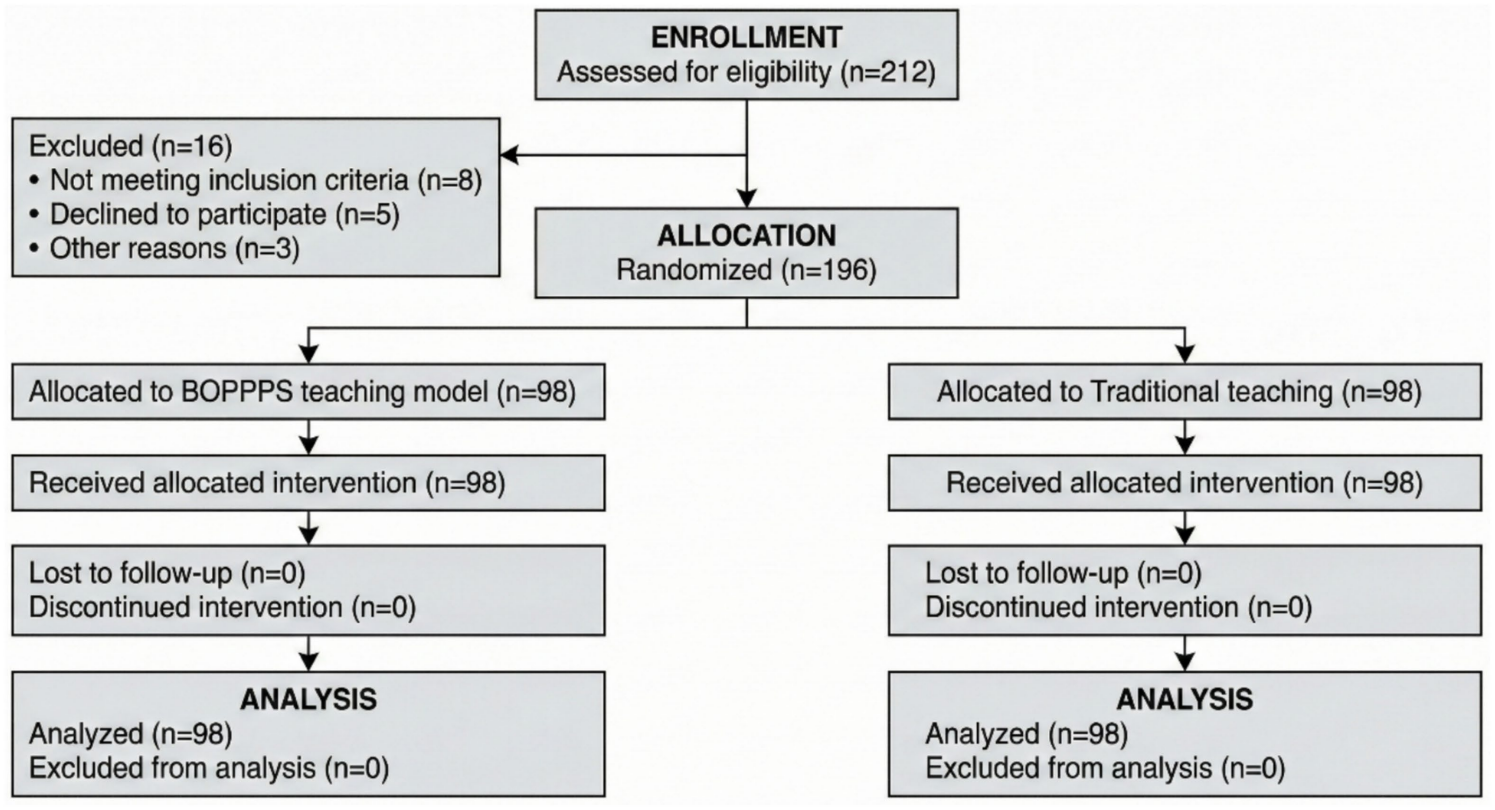
Flowchart of participant recruitment and study procedure (CONSORT style).

A total of 212 vascular surgery residents were initially screened for eligibility. After applying inclusion and exclusion criteria, 196 residents were enrolled and completed the study protocol. The participant recruitment and study flow are illustrated in Figure 1. Of the 16 residents excluded, 8 were from the experimental group (7 due to incomplete attendance, 1 withdrew from the program) and 8 were from the control group (6 due to incomplete attendance, 2 withdrew from the program).

As shown in Table 1, baseline demographic characteristics were comparable between the two groups. No significant differences were observed regarding age (experimental: 26.8 ± 2.4 years; control: 27.1 ± 2.6 years; *p* = 0.382), gender distribution (experimental: 62.2% male; control: 58.2% male; *p* = 0.556), educational background (*p* = 0.724), or training year (*p* = 0.831). Importantly, baseline SDLRS scores (142.35 ± 19.82 vs. 140.87 ± 20.45, *p* = 0.604) and baseline CTDI-CV scores (258.42 ± 27.36 vs. 255.18 ± 29.14, *p* = 0.425) were also comparable, indicating equivalent pre-intervention learning readiness and critical thinking disposition between groups.

**Table 1:** Baseline demographic characteristics of participants.

Variable	Experimental group (*n* = 98)	Control group (*n* = 98)	*p*-value
Age (years)	26.8 ± 2.4	27.1 ± 2.6	0.382
Gender (male, %)	61 (62.2%)	57 (58.2%)	0.556
Master’s degree (%)	72 (73.5%)	70 (71.4%)	0.724
Training year 2 (%)	48 (49.0%)	50 (51.0%)	0.831
Baseline SDLRS	142.35 ± 19.82	140.87 ± 20.45	0.604
Baseline CTDI-CV	258.42 ± 27.36	255.18 ± 29.14	0.425

### 3.2 Theoretical examination scores

**Figure 2:**
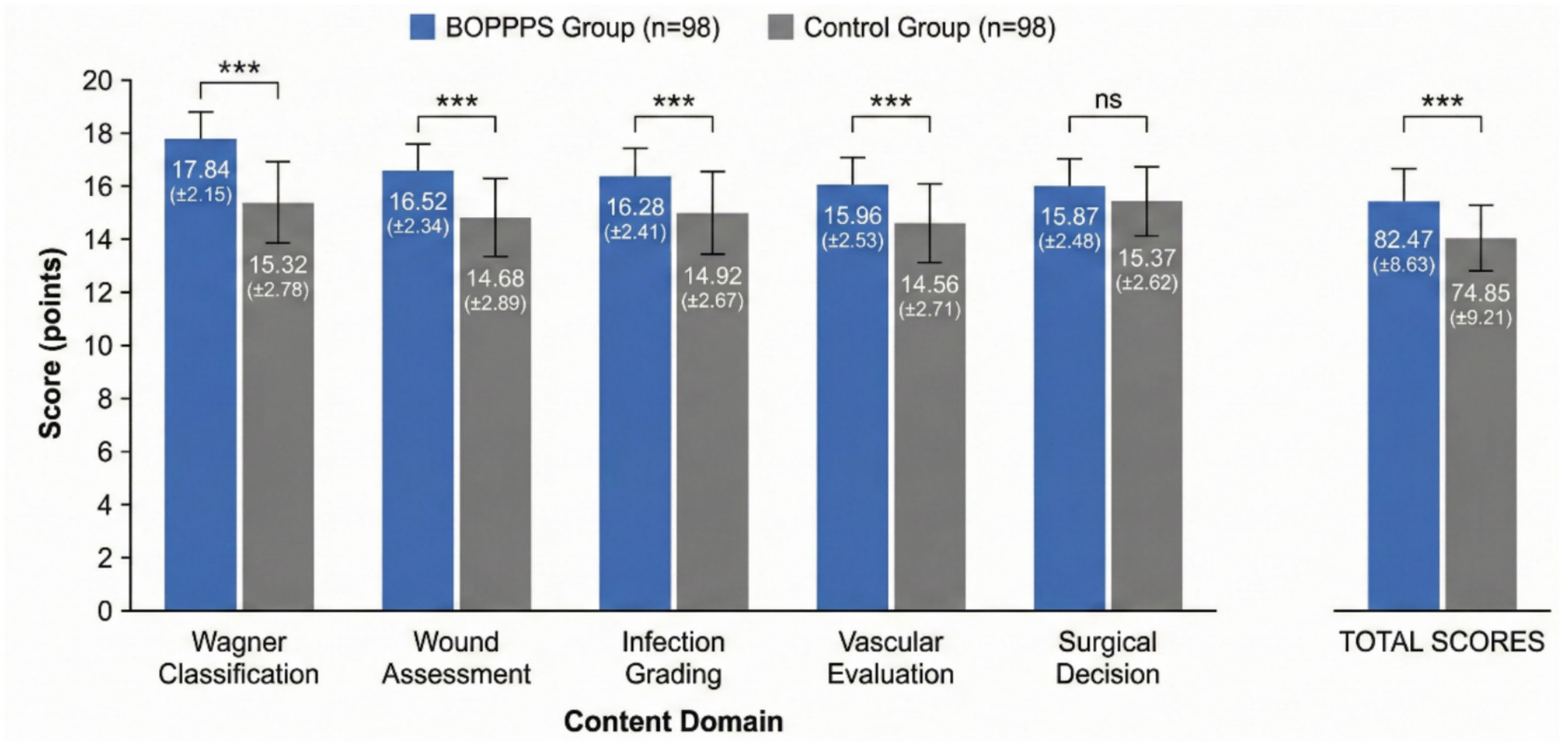
Comparison of theoretical examination scores between BOPPPS and traditional teaching groups. Error bars represent standard deviation. ***p < 0.001 compared to control group.

The comparison of theoretical examination scores between groups is presented in Table 2 and visually depicted in Figure 2. The experimental group achieved significantly higher total theoretical examination scores compared to the control group (82.47 ± 8.63 vs. 74.85 ± 9.21, *p* < 0.001, Cohen’s *d* = 0.85). Sub-analysis across content domains revealed that the BOPPPS group performed significantly better in Wagner classification, wound assessment, infection grading, and vascular evaluation (all *p* < 0.001). However, the difference in surgical decision-making scores was not statistically significant (*p* = 0.173).

**Table 2:** Comparison of theoretical examination scores.

Content domain	Experimental group	Control group	*p*-value	Cohen’s *d*
Wagner classification	17.84 ± 2.15	15.32 ± 2.78	<0.001	1.02
Wound assessment	16.52 ± 2.34	14.68 ± 2.89	<0.001	0.70
Infection grading	16.28 ± 2.41	14.92 ± 2.67	<0.001	0.53
Vascular evaluation	15.96 ± 2.53	14.56 ± 2.71	<0.001	0.53
Surgical decision	15.87 ± 2.48	15.37 ± 2.62	0.173	0.20
Total score	82.47 ± 8.63	74.85 ± 9.21	<0.001	0.85

### 3.3 Clinical competence assessment

**Figure 3:**
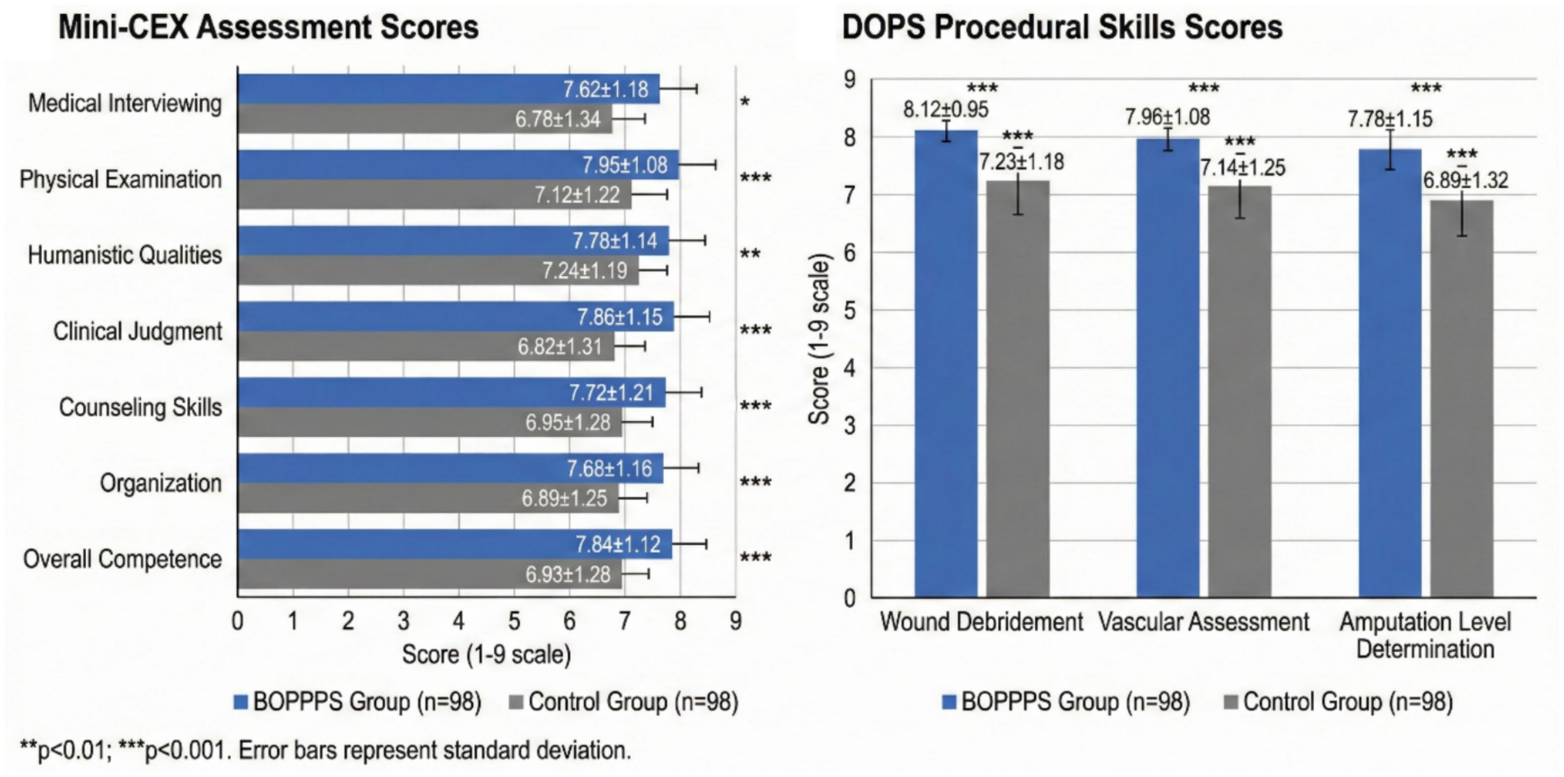
Comparison of Mini-CEX overall scores and DOPS procedural scores between BOPPPS and traditional teaching groups. Error bars represent standard deviation. ***p < 0.001 compared to control group.

Clinical competence was evaluated using Mini-CEX and DOPS assessments, with results summarized in Tables 3, 4 and Figure 3. Mini-CEX assessments demonstrated significantly higher scores in the experimental group across all seven evaluated domains. As detailed in Table 3, the experimental group achieved superior performance in medical interviewing skills (7.62 ± 1.18 vs. 6.78 ± 1.34, *p* < 0.001), physical examination skills (7.95 ± 1.08 vs. 7.12 ± 1.22, *p* < 0.001), humanistic qualities (7.78 ± 1.14 vs. 7.24 ± 1.19, *p* = 0.002), clinical judgment (7.86 ± 1.15 vs. 6.82 ± 1.31, *p* < 0.001), counseling skills (7.72 ± 1.21 vs. 6.95 ± 1.28, *p* < 0.001), organization/efficiency (7.68 ± 1.16 vs. 6.89 ± 1.25, *p* < 0.001), and overall clinical competence (7.84 ± 1.12 vs. 6.93 ± 1.28, *p* < 0.001, Cohen’s *d* = 0.76).

**Table 3:** Mini-CEX assessment results.

Domain	Experimental group	Control group	*p*-value	Cohen’s *d*
Medical interviewing	7.62 ± 1.18	6.78 ± 1.34	<0.001	0.67
Physical examination	7.95 ± 1.08	7.12 ± 1.22	<0.001	0.72
Humanistic qualities	7.78 ± 1.14	7.24 ± 1.19	0.002	0.46
Clinical judgment	7.86 ± 1.15	6.82 ± 1.31	<0.001	0.84
Counseling skills	7.72 ± 1.21	6.95 ± 1.28	<0.001	0.62
Organization	7.68 ± 1.16	6.89 ± 1.25	<0.001	0.65
Overall competence	7.84 ± 1.12	6.93 ± 1.28	<0.001	0.76

**Table 4:** DOPS assessment results.

Procedure	Experimental group	Control group	*p*-value	Cohen’s *d*
Wound debridement	8.12 ± 0.95	7.23 ± 1.18	<0.001	0.83
Vascular assessment	7.96 ± 1.08	7.14 ± 1.25	<0.001	0.70
Amputation level determination	7.78 ± 1.15	6.89 ± 1.32	<0.001	0.72

DOPS assessments for three key diabetic foot-related procedures demonstrated consistent improvements in the experimental group, as shown in Table 4. Wound debridement technique scores were significantly higher in the BOPPPS group (8.12 ± 0.95 vs. 7.23 ± 1.18, *p* < 0.001, Cohen’s *d* = 0.83). Similarly, vascular assessment skills including ankle-brachial index measurement showed significant improvement (7.96 ± 1.08 vs. 7.14 ± 1.25, *p* < 0.001, Cohen’s *d* = 0.70). Amputation level determination, a critical decision-making skill in diabetic foot surgery, also demonstrated significant enhancement in the experimental group (7.78 ± 1.15 vs. 6.89 ± 1.32, *p* < 0.001, Cohen’s *d* = 0.72). Figure 3 provides a visual comparison of both Mini-CEX and DOPS scores between groups.

### 3.4 Self-directed learning readiness

Changes in self-directed learning readiness, as measured by the SDLRS, are presented in Table 5. Post-intervention SDLRS total scores were significantly higher in the experimental group (168.52 ± 18.74) compared to the control group (153.28 ± 21.36, *p* < 0.001). Analysis of individual subscales revealed significant between-group differences in self-management (56.84 ± 7.23 vs. 51.36 ± 8.45, *p* < 0.001), desire for learning (58.92 ± 6.87 vs. 53.14 ± 7.92, *p* < 0.001), and self-control (52.76 ± 7.14 vs. 48.78 ± 7.68, *p* < 0.001). Within-group analysis demonstrated that the experimental group showed a substantial improvement from baseline (*Δ* = 26.17 ± 12.35, paired *t*-test *p* < 0.001), whereas the control group exhibited only modest improvement (Δ = 12.41 ± 14.28, paired *t*-test *p* = 0.023). The between-group difference in change scores was statistically significant (*p* < 0.001).

**Table 5:** Self-directed learning readiness scale scores.

Subscale	Experimental group (post)	Control group (post)	*p*-value
Self-management	56.84 ± 7.23	51.36 ± 8.45	<0.001
Desire for learning	58.92 ± 6.87	53.14 ± 7.92	<0.001
Self-control	52.76 ± 7.14	48.78 ± 7.68	<0.001
Total score	168.52 ± 18.74	153.28 ± 21.36	<0.001
Change from baseline (Δ)	26.17 ± 12.35	12.41 ± 14.28	<0.001

### 3.5 Critical thinking disposition

**Figure 4:**
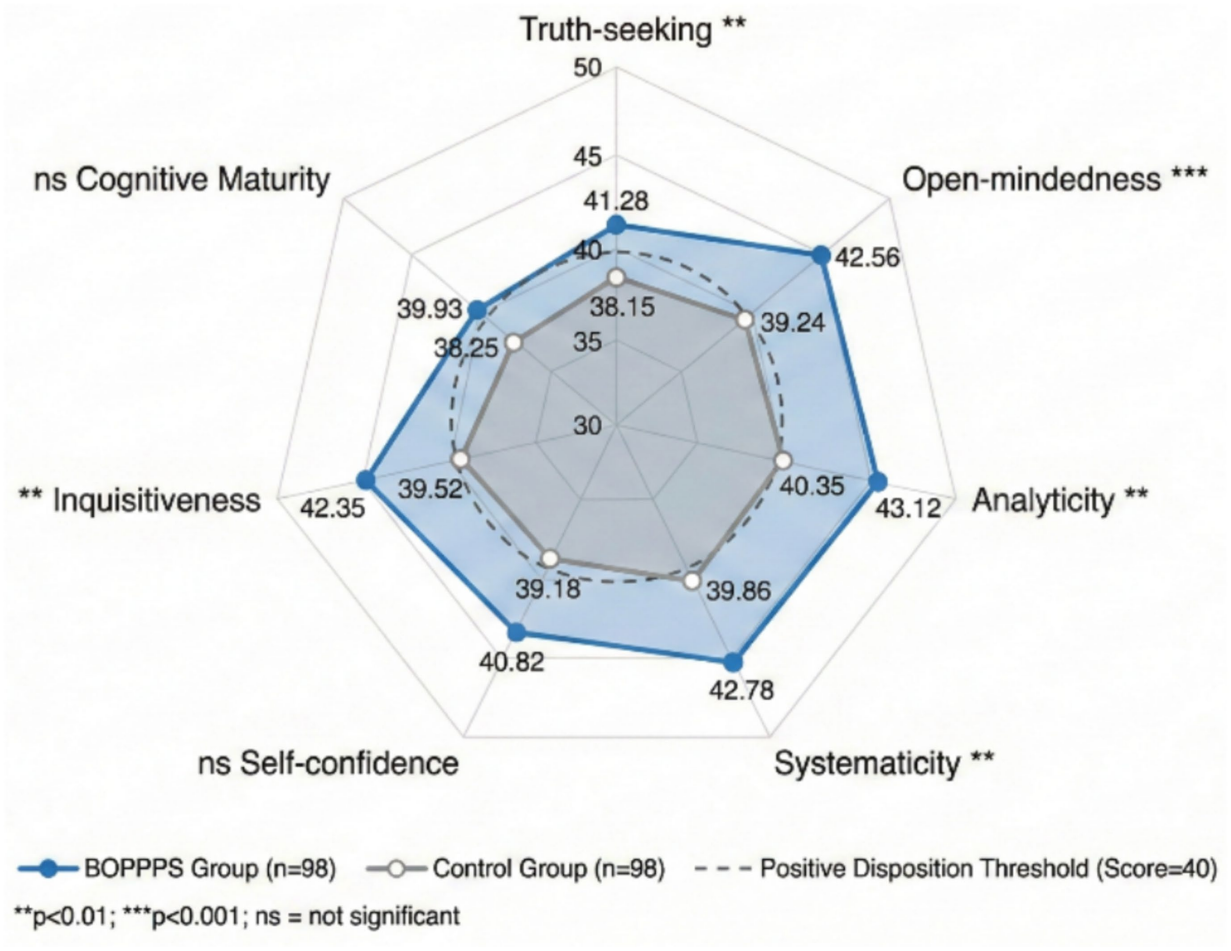
Radar chart comparing CTDI-CV subscale scores between BOPPPS and traditional teaching groups. The dashed line indicates the threshold for positive disposition (score = 40). *p < 0.05, **p < 0.01, ***p < 0.001 compared to control group.

Critical thinking disposition outcomes are detailed in Table 6 and illustrated in Figure 4. Post-intervention CTDI-CV total scores were significantly higher in the experimental group (292.84 ± 28.65) compared to the control group (271.35 ± 31.42, *p* < 0.001). As shown in Figure 4, subscale analysis revealed that the BOPPPS group demonstrated significantly better performance in truth-seeking (41.28 ± 6.24 vs. 38.15 ± 6.82, *p* = 0.001), open-mindedness (42.56 ± 5.87 vs. 39.24 ± 6.35, *p* < 0.001), analyticity (43.12 ± 5.64 vs. 40.35 ± 6.12, *p* = 0.001), systematicity (42.78 ± 5.92 vs. 39.86 ± 6.28, *p* = 0.002), and inquisitiveness (42.35 ± 5.78 vs. 39.52 ± 6.14, *p* = 0.001). However, differences in self-confidence (40.82 ± 6.35 vs. 39.18 ± 6.52, *p* = 0.078) and cognitive maturity (39.93 ± 6.18 vs. 38.25 ± 6.45, *p* = 0.064) did not reach statistical significance. Notably, 71.4% of residents in the experimental group achieved CTDI-CV scores ≥280 (indicating positive critical thinking disposition), compared to 52.0% in the control group (*χ*^2^ = 7.86, *p* = 0.005).

**Table 6:** Critical thinking disposition inventory scores.

Subscale	Experimental group	Control group	*p*-value
Truth-seeking	41.28 ± 6.24	38.15 ± 6.82	0.001
Open-mindedness	42.56 ± 5.87	39.24 ± 6.35	<0.001
Analyticity	43.12 ± 5.64	40.35 ± 6.12	0.001
Systematicity	42.78 ± 5.92	39.86 ± 6.28	0.002
Self-confidence	40.82 ± 6.35	39.18 ± 6.52	0.078
Inquisitiveness	42.35 ± 5.78	39.52 ± 6.14	0.001
Cognitive maturity	39.93 ± 6.18	38.25 ± 6.45	0.064
Total score	292.84 ± 28.65	271.35 ± 31.42	<0.001

### 3.6 Teaching satisfaction

**Figure 5:**
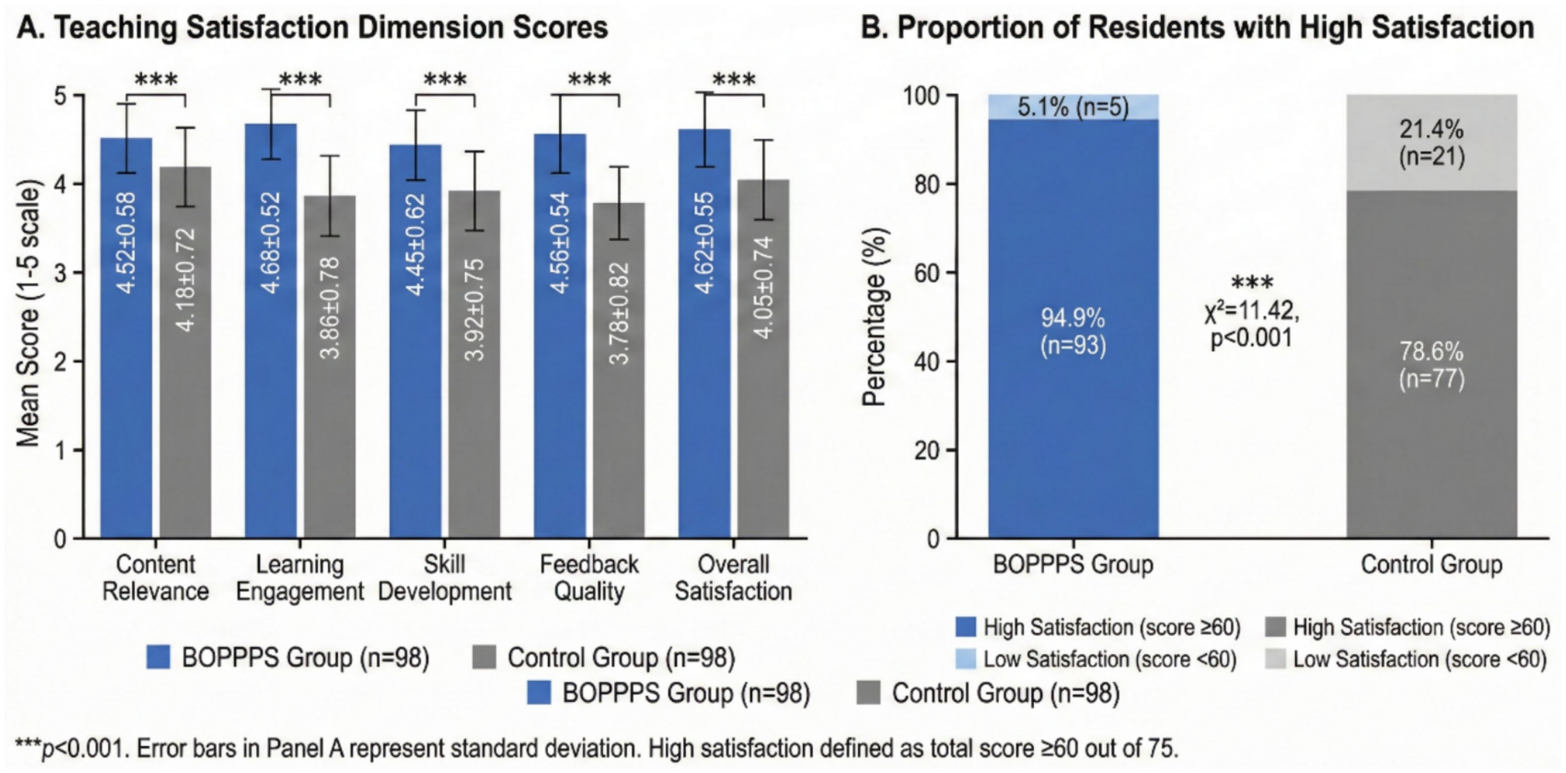
Comparison of teaching satisfaction between BOPPPS and traditional teaching groups. **(A)** Satisfaction dimension scores (mean ± SD). **(B)** Proportion of residents reporting high satisfaction (score ≥60). ***p < 0.001 compared to control group.

Teaching satisfaction outcomes are presented in Table 7 and Figure 5. Overall, 93 residents (94.9%) in the experimental group reported high satisfaction (total score ≥60) compared to 77 residents (78.6%) in the control group, representing a statistically significant difference (χ^2^ = 11.42, *p* < 0.001). As illustrated in Figure 5, analysis of specific satisfaction dimensions revealed that the BOPPPS group reported significantly higher satisfaction across all measured domains: content relevance (4.52 ± 0.58 vs. 4.18 ± 0.72, *p* < 0.001), learning engagement (4.68 ± 0.52 vs. 3.86 ± 0.78, *p* < 0.001), skill development (4.45 ± 0.62 vs. 3.92 ± 0.75, *p* < 0.001), feedback quality (4.56 ± 0.54 vs. 3.78 ± 0.82, *p* < 0.001), and overall satisfaction (4.62 ± 0.55 vs. 4.05 ± 0.74, *p* < 0.001). The largest between-group difference was observed in learning engagement (Cohen’s *d* = 1.24), suggesting that the interactive nature of BOPPPS-based instruction was particularly valued by residents.

**Table 7:** Teaching satisfaction results.

Satisfaction dimension	Experimental group	Control group	*p*-value	Cohen’s *d*
Content relevance	4.52 ± 0.58	4.18 ± 0.72	<0.001	0.52
Learning engagement	4.68 ± 0.52	3.86 ± 0.78	<0.001	1.24
Skill development	4.45 ± 0.62	3.92 ± 0.75	<0.001	0.77
Feedback quality	4.56 ± 0.54	3.78 ± 0.82	<0.001	1.12
Overall satisfaction	4.62 ± 0.55	4.05 ± 0.74	<0.001	0.88
High satisfaction rate (%)	93 (94.9%)	77 (78.6%)	<0.001	—

## 4 Discussion

The present study provides evidence that the BOPPPS teaching model can be effectively implemented in the context of standardized training for vascular surgery residents. Residents receiving BOPPPS-based instruction demonstrated superior outcomes in theoretical knowledge, clinical competence, self-directed learning readiness, critical thinking disposition, and teaching satisfaction compared to those receiving traditional teaching. These findings contribute to the growing body of evidence supporting structured, learner-centered pedagogical approaches in specialized medical education.

The substantial improvement in theoretical examination scores observed in the BOPPPS group aligns with findings from previous studies examining this teaching model in various medical education contexts. Ma et al. conducted a systematic review and meta-analysis demonstrating that BOPPPS-based instruction consistently improved academic performance across diverse medical disciplines (14). The structured nature of the BOPPPS framework, with explicit learning objectives and systematic assessment components, appears to facilitate knowledge acquisition and retention more effectively than traditional lecture-based approaches. In the context of diabetic foot education, the pre-assessment component allowed instructors to identify baseline knowledge gaps, while participatory learning activities promoted active engagement with complex classification criteria and treatment algorithms (17).

The superior clinical competence demonstrated by BOPPPS-trained residents, as evidenced by Mini-CEX and DOPS scores, represents a particularly meaningful finding given the procedural nature of vascular surgery. Previous research has established that workplace-based assessments including Mini-CEX and DOPS provide valid measures of clinical performance and are increasingly utilized in residency training programs worldwide (18). The improvement in clinical judgment scores observed in our study suggests that the case-based discussions and standardized patient simulations incorporated within the participatory learning component effectively bridged theoretical knowledge and clinical application. Similar findings were reported by Hu et al., who demonstrated that BOPPPS-based thoracic surgery education improved residents’ clinical decision-making abilities (19).

The significant enhancement of self-directed learning readiness in the BOPPPS group has important implications for lifelong professional development. Self-directed learning represents a fundamental competency for physicians, enabling continuous knowledge updating in response to rapidly evolving medical practice (20). The BOPPPS model promotes learner autonomy through its emphasis on clear objectives, self-assessment opportunities, and active participation. Our findings are consistent with those of Li et al. (21), who reported that BOPPPS combined with team-based learning improved self-directed learning abilities in nursing students. The pre-assessment and post-assessment components of BOPPPS appear particularly valuable in fostering metacognitive skills essential for self-directed learning.

The improvement in critical thinking disposition among BOPPPS-trained residents addresses an important educational goal that has received increasing attention in medical education. Critical thinking enables physicians to analyze complex clinical scenarios, evaluate evidence, and make sound decisions under uncertainty (22). The case-based discussions and problem-solving activities embedded within the BOPPPS participatory learning component require residents to apply analytical skills, consider multiple perspectives, and justify clinical reasoning. Wang et al. demonstrated that medical students’ critical thinking disposition was positively associated with problem-based learning performance (23), supporting the pedagogical value of active learning strategies incorporated in the BOPPPS model.

The high levels of teaching satisfaction reported by residents in the BOPPPS group suggest that this instructional approach is well-received by learners. Satisfaction with educational experiences may influence learning motivation, engagement, and ultimately educational outcomes (24). The interactive nature of BOPPPS-based instruction, combined with regular feedback opportunities, appears to create a more engaging and supportive learning environment compared to traditional didactic approaches. Previous studies examining BOPPPS in obstetrics and gynecology, nephrology, and intensive care medicine have similarly reported high satisfaction rates among trainees (25).

Several practical implications emerge from our findings. First, vascular surgery residency programs should consider implementing structured pedagogical frameworks such as BOPPPS for teaching complex topics requiring integration of theoretical knowledge and clinical skills. Second, the success of BOPPPS implementation depends on adequate faculty development to ensure instructors are proficient in designing and delivering interactive learning activities. Third, the incorporation of workplace-based assessments including Mini-CEX and DOPS provides valuable formative feedback to guide resident development. Finally, attention to self-directed learning and critical thinking development should be explicitly incorporated into residency curricula to prepare residents for lifelong learning.

This study has several limitations that should be acknowledged. First, as this was a quasi-experimental study without randomization, causal inference is limited. Although baseline characteristics were comparable and allocation was based on rotation schedule to minimize disruption, unmeasured confounding variables cannot be entirely ruled out. Second, the study was conducted at a single institution, which may limit generalizability to other training contexts. Third, long-term retention of knowledge and skills was not assessed, and the durability of observed improvements remains unknown. Fourth, while the same pool of faculty instructors taught both groups, we cannot exclude potential instructor-related effects, such as differences in enthusiasm or engagement when delivering the novel BOPPPS curriculum. Fifth, the assessments were conducted by faculty evaluators who, while blinded to group allocation, may have been subject to observer bias. Finally, the absence of long-term follow-up means we cannot determine whether the improvements in critical thinking and self-directed learning translate into sustained clinical competency or improved patient outcomes. Future prospective, multicenter, randomized controlled trials with longer follow-up periods and more objective outcome measures are warranted to confirm and extend these findings.

This finding is noteworthy as it highlights a potential limitation of short-term educational interventions. Surgical decision-making is a complex competency that integrates not only knowledge of classification systems and treatment algorithms but also experiential learning, pattern recognition, and the ability to weigh risks and benefits under uncertainty (22). While the BOPPPS model enhanced foundational knowledge, the two-week intervention may have been insufficient for residents to fully develop and internalize this advanced cognitive skill. This interpretation is supported by the non-significant improvements in the ‘self-confidence’ and ‘cognitive maturity’ subscales of the CTDI-CV, which are integral to sound clinical judgment. It is plausible that these dispositional attributes, which underpin confident and mature decision-making, require more prolonged and varied clinical exposure to develop (23). Future curriculum designs should consider integrating spaced learning, repeated case-based simulations, and longitudinal mentoring to specifically target and reinforce the development of higher-order clinical decision-making skills.

In conclusion, this study provides evidence that the BOPPPS teaching model represents an effective pedagogical approach for standardized training of vascular surgery residents in diabetic foot Wagner classification and surgical intervention strategies. The significant improvements observed in theoretical knowledge, clinical competence, self-directed learning readiness, critical thinking disposition, and teaching satisfaction support the broader implementation of this structured instructional framework in vascular surgery education. As the burden of diabetic foot disease continues to increase globally, ensuring that vascular surgery residents are optimally prepared to manage this complex condition remains a critical educational priority.

## Data Availability

The original contributions presented in the study are included in the article/supplementary material, further inquiries can be directed to the corresponding author.
